# Analgesic, antiallodynic, and anticonvulsant activity of novel hybrid molecules derived from *N*-benzyl-2-(2,5-dioxopyrrolidin-1-yl)propanamide and 2-(2,5-dioxopyrrolidin-1-yl)butanamide in animal models of pain and epilepsy

**DOI:** 10.1007/s00210-017-1358-3

**Published:** 2017-02-10

**Authors:** Anna Rapacz, Krzysztof Kamiński, Jolanta Obniska, Paulina Koczurkiewicz, Elżbieta Pękala, Barbara Filipek

**Affiliations:** 1 Department of Pharmacodynamics, Faculty of Pharmacy, Jagiellonian University Medical College, Medyczna 9, 30-688 Krakow, Poland; 2 Department of Medicinal Chemistry, Faculty of Pharmacy, Jagiellonian University Medical College, Medyczna 9, 30-688 Krakow, Poland; 3 Department of Pharmaceutical Biochemistry, Faculty of Pharmacy, Jagiellonian University Medical College, Medyczna 9, 30-688 Krakow, Poland

**Keywords:** Hot plate, Formalin, Oxaliplatin, Psychomotor seizures, Binding studies, Hybrid compounds

## Abstract

The purpose of the present study was to examine the analgesic activity of six novel hybrid molecules, which demonstrated in the previous research anticonvulsant activity in the maximal electroshock seizure (MES) and subcutaneous pentylenetetrazole seizure (scPTZ) tests in mice. The antinociceptive properties were estimated in three models of pain in mice—the hot plate test, the formalin test, and in the oxaliplatin-induced neuropathy. Moreover, extended anticonvulsant studies were carried out and the antiseizure activity was investigated in the 6-Hz test. Considering drug safety evaluation, the influence of compounds on locomotor activity and contextual memory were checked. Furthermore, chosen molecules were tested in vitro for potential hepatotoxicity. To explain the probable mechanism of action, the radioligand binding assays were performed. In both phases of formalin test, analgesic activity demonstrated compounds **4**, **8**, and **9**. These agents relieved also mechanical allodynia in oxaliplatin-induced model of neuropathic pain. At active doses, they did not influence locomotor activity of mice. Moreover, for compounds **8** and **9**, no deleterious effect on memory was observed, but compound **4** might induce memory deficits. All tested compounds (**4**, **5**, **8**, **9**, **15**, and **16**) inhibited psychomotor seizures with the ED_50_ values = 24.66–47.21 mg/kg. The binding studies showed that compound **4** only at the high concentrations revealed the effective binding to the neuronal sodium channels and moderately binding to the L-type calcium (verapamil site) channels and NMDA receptors. The present preclinical results proved that novel hybrid molecules demonstrate very promising anticonvulsant and analgesic activity.

## Introduction

Epilepsy is a chronic neurological disorder that is associated with various types of recurrent convulsive and non-convulsive seizures. Although 70–80% of the patients with new onset epilepsy have complete seizure control with current antiepileptic drugs, more effective, better-tolerated treatments remains major unmet need (Sidhu and Sadhotra 2016). Currently available first- and second-generation anticonvulsant drugs are known to cause multiple adverse effects, including dizziness, diplopia, ataxia, and drowsiness; therefore, they had a significant impact on quality of life of people with epilepsy (Cramer et al. 2010).

Most anticonvulsant drugs were developed to treat epilepsy, and their therapeutic activity in other disorders, including pain (neuropathic pain, migraine prophylaxis), neuromuscular, and bipolar disorders was proved later (Mantegazza et al. 2010). Evidence suggests that epilepsy and pain syndromes have common pathophysiological mechanisms. One of them is hyperexcitability generated by the pathological expression and redistribution of sodium and calcium channels. Some of anticonvulsant drugs that block these channels are effective in the treatment of neuropathic pain owing to the same selective block of high-frequency action potential firing that accounts for their protective activity against seizures. Among these drugs, carbamazepine was found to be useful in the treatment of trigeminal neuralgia, whereas the gabapentinoids (like gabapentin and pregabalin) were efficacious drugs in diabetic neuropathic pain and post-herpetic neuralgia (Kukkar et al. 2013; Mendlik and Uritsky 2015).

Taking into consideration the aforementioned facts, continued preclinical searching for new anticonvulsant drugs with collateral antinociceptive activity are expected since they lead to further advancements in the treatment of epilepsy as well as neuropathic pain.

In the recent study, we demonstrated significant anticonvulsant activity of six new hybrid compounds in two classic animal models of epilepsy, MES and scPTZ tests (Kamiński et al. 2015a). These two models MES and scPTZ tests are well known as “gold standards” in preclinical studies for early detection of anticonvulsant activity (Löscher and Schmidt 2011). Because of the increasing role of anticonvulsant drugs for treatment of neuropathic pain, studies on the search for new anticonvulsant agents should also consider the evaluation of their usefulness in the treatment of this type of neurological disorder (Rogawski and Löscher 2004). Therefore, the first aim of the present study was to evaluate analgesic activity of the selected compounds. The antinociceptive properties were estimated in the hot plate test of acute pain, the formalin model of persistent pain, as well as in the oxaliplatin-induced neuropathic pain model in mice. Looking for the new compounds with broad spectrum of activity in animal models of epilepsy, the second aim of the present experiments was to extend our anticonvulsant studies: tested compounds were also examined in the 6 Hz model of pharmacoresistant limbic seizures. Moreover, the influence on spontaneous locomotor activity as well as cognition in passive avoidance test were checked. Considering drug safety evaluation, which is important in the preclinical identification of new active substances, they were tested for potential hepatotoxicity on human hepatocellular carcinoma cell line using in vitro cellular model. To determine the probable mechanism of anticonvulsant action for the chosen compound, in vitro ion channels and receptor binding assays were carried out.

## Materials and methods

### Animals

All experiments were carried out on adult male CD-1 mice (22–26 g). The animals were housed in plastic cages at room temperature of 20 ± 2 °C under 12–12 h light-dark cycle. A standard pellet diet and tap water were continuously available. All the experiments were performed between 8 a.m. and 3 p.m., after a minimum 30-min acclimatization to the experimental room. The animals were randomly assigned to the experimental groups and killed by cervical dislocation immediately after the experiment.

### Drugs and chemicals

**Scheme 1:**
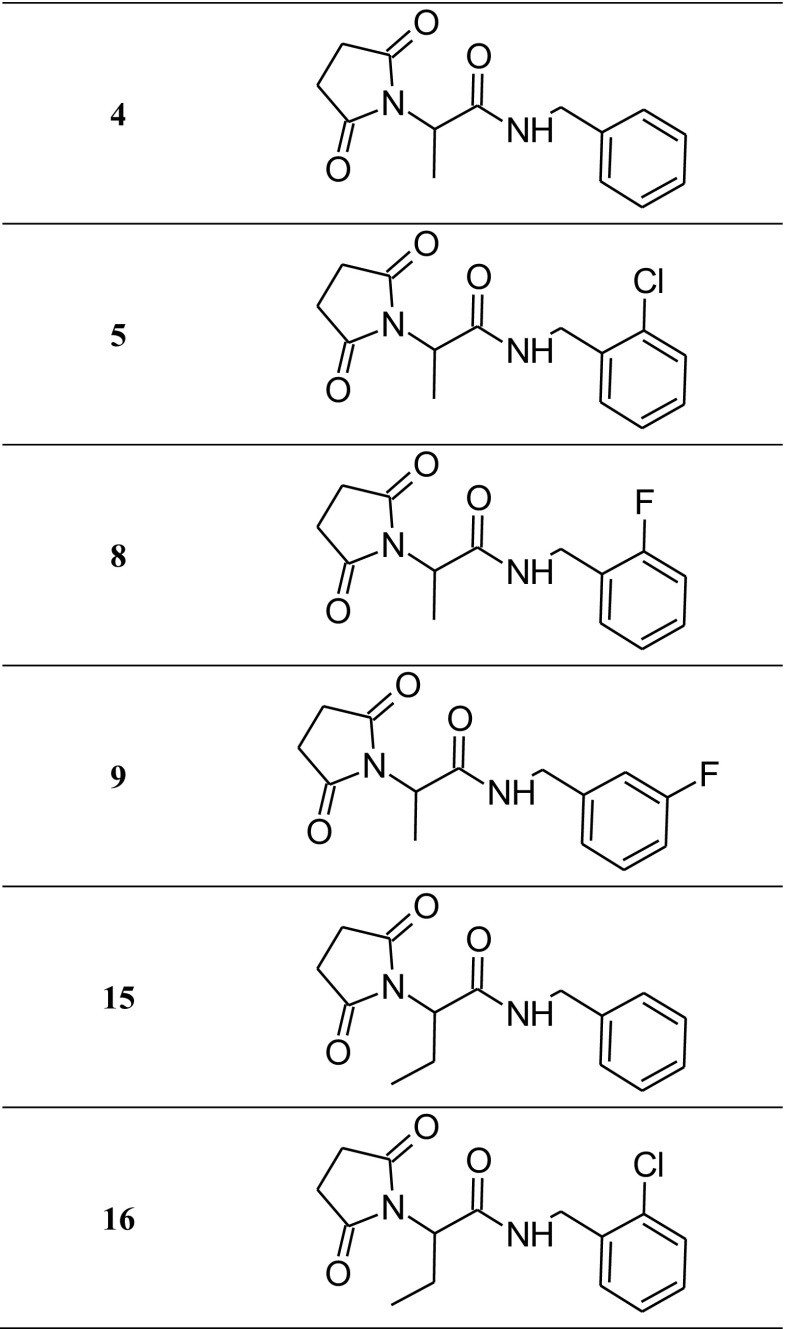
Schematic structure of the tested compounds

The investigated compounds, **4** (*N*-benzyl-2-(2,5-dioxopyrrolidin-1-yl)propanamide), **5** (*N*-(2-chlorobenzyl)-2-(2,5-dioxopyrrolidin-1-yl)propanamide), **8** (2-(2,5-dioxopyrrolidin-1-yl)-*N*-(2-fluorobenzyl)propanamide), **9** (2-(2,5-dioxopyrrolidin-1-yl)-*N*-(3-fluorobenzyl)propanamide), **15** (*N*-benzyl-2-(2,5-dioxopyrrolidin-1-yl)butanamide), and **16** (*N*-(2-chlorobenzyl)-2-(2,5-dioxopyrrolidin-1-yl)butanamide) were synthesized at the Department of Medicinal Chemistry, Jagiellonian University, Medical College in Krakow (Scheme 1). The synthesis and preliminary pharmacological studies of the investigated compounds was presented in our previous study (Kamiński et al. 2015a). For the in vivo experiments, the tested agents were suspended in a 0.5% solution of methylcellulose (Loba Chemie, Germany). Lacosamide (Vimpat, UCB Pharma, Belgium), levetiracetam (Sigma-Aldrich, Germany), and valproic acid (Sigma-Aldrich, Poland) were dissolved in saline solution. Formaldehyde (POCH, Poland) was dissolved in distilled water. Oxaliplatin (Tocris Bioscience, UK) was prepared in a 5% aqueous solution of glucose. All drug solutions/suspensions were prepared freshly and given intraperitoneally (i.p.) in a volume of 10 ml/kg. The tested compounds were examined at the dose which was its median effective dose (ED_50_) determined in the MES test in the previous study (Table 1) (Kamiński et al. 2015a). In the initial anticonvulsant evaluations in the 6-Hz test, the animals were administered with a constant dose of 100 mg/kg of each compound and experiments were carried out 0.25, 0.5, 1, and 2 h after i.p. injection (Rapacz et al. 2016a). Then the ED_50_ values were established at previously estimated time of peak effect. Reference drugs were administered as follows: lacosamide and valproic acid, 30 min, and levetiracetam, 60 min, before the tests. The pretreatment times before the testing of reference anticonvulsant drugs were based upon information about their biological activity from the literature and our previous experiments (Rapacz et al. 2016b; Rybka et al. 2016).

**Table 1:** Dose of tested or references compounds which was used in the present study (ED_50_ values determined in the MES test)

Compound	Dose (mg/kg)
**4**	67.65
**5**	74.49
**8**	54.90
**9**	77.43
**15**	73.26
**16**	113.02
Valproic acid	252.74
Lacosamide	9.24

### Hot plate test

Antinociceptive activity in the hot plate test (model of acute pain) was investigated as described previously (Rapacz et al. 2016b) using the hot plate apparatus (Hot/cold plate, Bioseb, France). In this experiment, the latency to pain reaction (hind paw licking or jumping) of mice to a nociceptive stimulus (55–56 °C) was recorded. The cutoff time was established to 60 s to avoid paw tissue damage.

### Formalin test

The formalin test was performed according to Laughlin et al. (2002). The experimental procedure of this model of tonic pain was described in detail in our earlier studies (Rapacz et al. 2016a; Rapacz et al. 2016b). Briefly, 20 μl of a 2.5% formalin solution was injected into the dorsal surface of the right hind paw of each mouse. Then, the animals were placed individually into glass beakers and were observed for the next 30 min. Time spent on licking or biting the injected hind paw in selected intervals, 0–5 (the acute nociceptive phase) and 15–30 min (the inflammatory phase), was measured in each experimental group.

### Spontaneous locomotor activity

Locomotor activity was recorded with photoresistor actometers (Ugo Basile, Italy) as described previously (Rapacz et al. 2016b). The tested or reference compounds were administered i.p. at the doses corresponding to their ED_50_ values from the MES test, which were obtained in our previous studies (Kamiński et al. 2015a; Rybka et al. 2014) to determine whether the compounds at active anticonvulsant dose influence normal locomotor activity of mice. Mice were individually placed in activity cages (40 × 40 × 30 cm) for a 30-min habituation period, and then the number of light-beam crossings was counted during the next 30 min of the experiment.

### Oxaliplatin-induced neuropathic pain

Anitallodynic activity was examined in the model of neuropathic pain induced by oxaliplatin as described in detail earlier (Rapacz et al. 2016a; Sałat et al. 2014). Mechanical withdrawal thresholds were examined for all mice prior to oxaliplatin injection to obtain normal baseline values of withdrawal threshold to mechanical stimuli (control). Seven days after post-oxaliplatin injection (10 mg/kg, i.p.), the effect of test compounds on mechanical allodynia was assessed using the electronic von Frey device (Panlab, Spain). Mice were placed individually in test compartments on an elevated metal mesh grid and allowed to acclimate for 30 min. After a habituation period, each mouse was tested three times in the midplantar region of hind paw, allowing at least 30 s between each measurement. Subsequently, the animals were pretreated with the examined agents or vehicle. Thirty minutes later, the animals were tested again and mean values of mechanical withdrawal threshold were determined for each mouse.

### Passive avoidance step-through test

The effect of the tested and reference compounds on learning abilities of mice was conducted using the passive avoidance apparatus (Panlab/Harvard Apparatus, Spain) according to the method described elsewhere (Łuszczki et al. 2008; Pytka et al. 2016). The mice underwent two separate trials: an acquisition trial (conditioning phase) and a retention trial (testing phase) 24 h later. Thirty minutes before the acquisition trial, each mouse was pretreated with the tested or reference compound at the dose corresponding to its ED_50_ value from the MES test. As a control, vehicle-treated (0.5% methylcellulose, i.p.) and scopolamine-treated mice (1 mg/kg, i.p.) were also used. Each animal was placed for 30 s in an illuminated light compartment, and after this exploration period, the guillotine gate between the light and the dark compartments was open. As far as the mice came into the dark compartment, the gate was closed and an electrical foot shock (0.2 mA for 2 s) was given through the grid floor. On the following day, each mouse was placed again in the white compartment and the latency time between gate opening and entry into the dark compartment was recorded for each animal. Mice, which avoided the dark compartment for 180 s (cut of latency), were considered to remember the foot shock from the acquisition trial. Better memory performance was indicated by longer latency to enter in the black compartment in the test phase than in the conditioning phase (Sałat et al. 2016).

### 6 Hz psychomotor seizures

The 6-Hz test was performed according to Brown et al. (1953) and Barton et al. (2001) as described in detail earlier (Rapacz et al. 2016a). Psychomotor seizures were induced via corneal stimulation (6 Hz, 32 mA, 0.2 ms rectangular pulse width, 3-s duration) using a constant current device (ECT Unit 57800, Ugo Basile, Italy). During the stimulation, mice were gently restrained and released into the observation cage immediately after current application. In the vehicle-treated mice, the procedure caused “stunned” posture associated with rearing, forelimb clonus, automatic movements, and twitching of the vibrissae and Straub tail. The main seizure end point was the duration of the immobility. Mice resuming normal behavior within 10 s from the stimulation were considered as protected (Kaminski et al. 2004; Leclercq and Kaminski 2015; Rapacz et al. 2016a). To evaluate the ED_50_, at least three groups of animals were injected with various doses of tested compounds. Each group consisted of six animals. The ED_50_ was defined as the dose of a drug protecting 50% of animals against the 6 Hz seizures.

### In vitro binding studies

The radioligand binding studies were performed commercially by Cerep (Celle I’Evescault, France). Selected compound **4** was evaluated in preparations from rat cerebral cortex (ion channel binding, NMDA receptor binding), human recombinant (CHO cells) (GABAA1 binding), or SH-SY5Y cells (human recombinant) (neuronal α4β2 nicotinic binding). [^3^H]Batrachotoxinin for sodium channel (Brown 1986), [^3^H]nitrendipine for L-type calcium channel (dihydropyridine site) (Gould et al. 1982), [^3^H]D888 for L-type calcium channel (verapamil site) (Reynolds et al. 1986), [^125^I]*ω*-conotoxin GVIA for N-type calcium channel (Wagner et al. 1988), [^3^H]CGP 39653 for NMDA receptor (Sills et al. 1991), [^3^H]muscimol for GABAA1 (alpha 1,beta 2,gamma 2) receptor (Wang 2001), and [^3^H]cytisine for neuronal α4β2 nicotinic receptor (Gopalakrishnan et al. 1996) were used as specific radioligands, respectively. Compound binding was expressed as a percentage of inhibition of the binding of a radioactively labelled ligand.

### Analysis of hepatotoxicity activity using in vitro cellular model

#### Cell culture

Human cancer cells (Hep G2) ATCC® 59195™ were used in the study. The cells were cultured in standard conditions (37 °C, 5% CO_2_), in MEM medium (Sigma-Aldrich), supplemented with 10% FBS (Sigma-Aldrich) and antibiotics (Sigma-Aldrich).

#### MTT test

The MTT assay (Cayman) was used to determine the cytotoxic effects of analyzed compounds. Briefly, the cells were seeded at a density of 1 × 10^5^ in 96-well plates. Following overnight culture, the cells were then treated with increasing doses of compounds (**4** and **8**) as well as doxorubicin (positive control) and incubated for 24 h. Following cell exposure to compounds for 24 h, 10 μl MTT reagent was added to each well and after 3 h of incubation (37 °C, 5% CO_2_), dark crystals (reduced formazan) appeared in the bottom of the wells. Next, the Crystal Dissolving Solution (Cayman) was added to each well. Then the optical density (OD) of each well was determined at 570 nm on plate reader (BioTek). The number of metabolically active living cells is directly proportional to the absorbance of the samples. The results are presented at diagram as the percentage of control condition ± SEM.

### Data analysis

The obtained results were statistically estimated using one-way analysis of variance (ANOVA), followed by Dunnett’s test or repeated measures ANOVA, followed by Bonferroni’s multiple comparison test. The results from passive avoidance task were assessed using Kruskal-Wallis analysis of variance, followed by Dunn’s multiple comparison test. Differences between groups were considered as significant if *p* < 0.05. The log-probit method described by Litchfield and Wilcoxon (1949) was used to establish ED_50_ values with the 95% confidence limits.

## Results

### Antinociceptive activity in the hot plate test

**Fig. 1:**
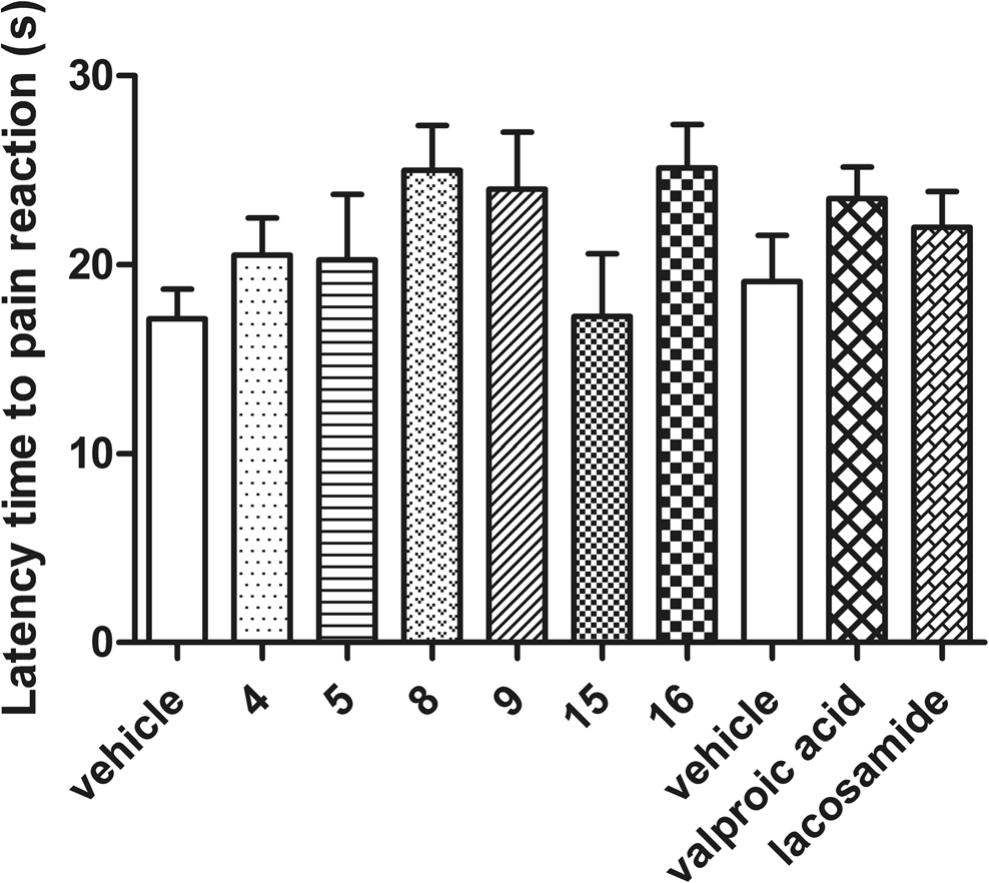
Effects of compounds **4**, **5**, **8**, **9**, **15**, **16**, valproic acid, and lacosamide on response latency in the hot plate test in mice. Data are presented as mean ± SEM. Statistical analysis of the results was conducted using one-way analysis of variance (ANOVA), followed by Dunnett’s test: *NS*, *N =* 8

As it was shown in Fig. 1 compounds **8**, **9**, and **16** prolonged the latency time to pain reaction from 17.4 ± 1.4 (vehicle-treated group) to 25.0 ± 2.3 (by 44%, comp. **8**), 24.0 ± 3.0 (by 38%, comp. **9**), and 25.1 ± 2.3 s (by 45%, comp. **16**), respectively, but not in a statistically significant way. Compounds **4** and **5** only slightly prolonged the latency to pain response, whereas compound **15** failed to induce analgesic action in this model of acute pain. Anticonvulsant drugs—valproic acid and lacosamide—prolonged the latency time to pain response from 19.1 ± 2.4 (vehicle-treated group) to 23.5 ± 1.7 (by 23%) and 22.0 ± 1.9 s (by 15%), respectively, but the results were not statistically significant.

### Analgesic activity in the formalin test

**Fig. 2:**
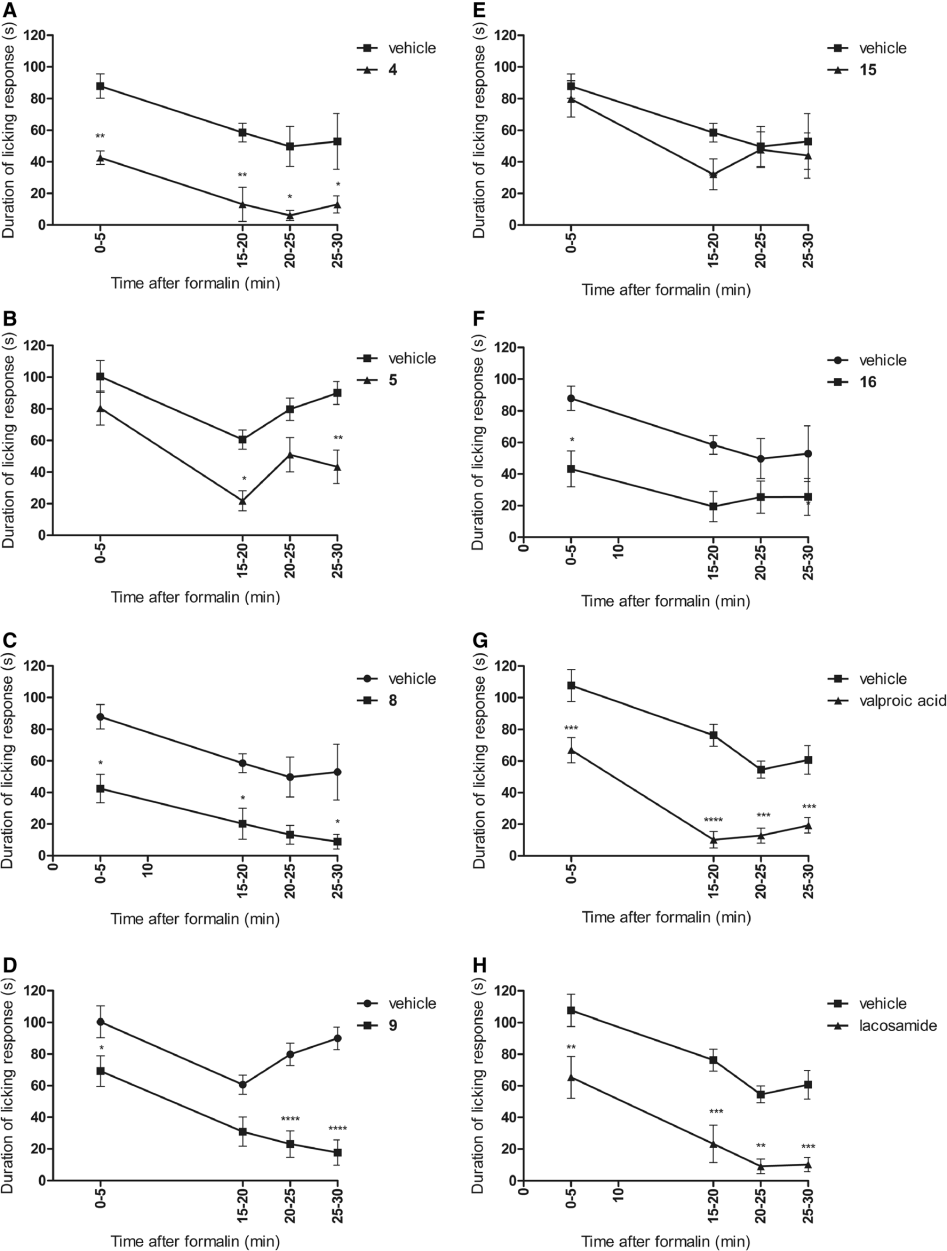
**a**–**h** Time course of the antinociceptive activity of compounds **4**, **5**, **8**, **9**, **15**, **16**, valproic acid, and lacosamide in the formalin test. Each value represents the mean ± SEM. Statistical analysis of the result was conducted using two-way repeated measures ANOVA, followed by Bonferroni’s multiple comparison test. Results compared to vehicle-treated mice at the same time points: *p < 0.05, **p < 0.01, ***p < 0.001, ****p < 0.0001, *N* = 8

As shown in Fig. 2a–f, in the first (neurogenic) phase of the test, four compounds, **4**, **8**, **9**, and **16**, significantly reduced the duration of the licking response by 51 (*p* < 0.01), 52 (*p* < 0.05), 31 (*p* < 0.05), and 51% (*p* < 0.05), respectively. In the second (inflammatory) phase of the formalin test, a statistically significant analgesic activity was recorded for four molecules: **4**, **5**, **8**, and **9**. The evaluation of the time course of the antinociceptive activity at selected intervals showed that only compound **4** strongly diminished the pain responses in all intervals: between 15 and 20 min of the observation by 78% (*p* < 0.01), between 20 and 25 min by 88% (*p* < 0.05), and between 25 and 30 min by 75% (*p* < 0.05). Compounds **5** and **8** attenuated the nocifensive response, in a statistically important way between 15 and 20 min of the measurement by 64 (*p* < 0.05) and 65% (*p* < 0.05), respectively, and in the last part of observation, between 25 and 30 min: **5** by 52% (*p* < 0.01) and **8** by 83% (*p* < 0.05). Compound **9** diminished the pain responses in a statistically significant way between 20 and 25 min of the assay by 71% (*p* < 0.0001) and between 25 and 30 min of the test by 80% (*p* < 0. 0001). In the inflammatory phase also, compound **16** showed analgesic action, but the results were not statistically significant. Compound **15** did not display antinociceptive properties in any phase of this model of tonic pain. Anticonvulsant drugs—valproic acid and lacosamide—displayed significant antinociceptive effect in this model of tonic pain, as they decreased the duration of the licking response in both phase: in the first phase by 38 (*p* < 0.001) and 39% (*p* < 0.01), respectively, and in the second phase in all intervals: between 15 and 20 min of the observation by 87 (*p* < 0.0001) and 70% (*p* < 0.001), between 20 and 25 min by 76 (*p* < 0.001) and 83% (*p* < 0.05), and between 25 and 30 min by 68 (*p* < 0.001) and 83% (*p* < 0.001), respectively (Fig. 2g, h).

### Influence on locomotor activity

**Fig. 3:**
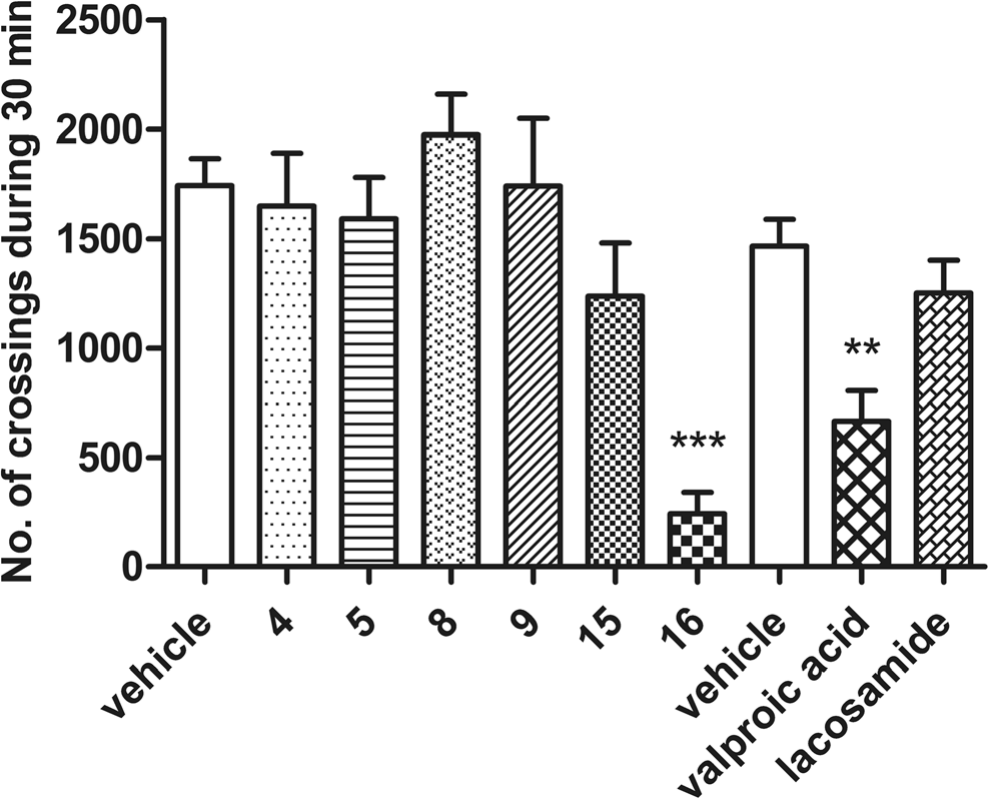
Effects of the tested and reference compounds on locomotor activity in mice. Data are presented as mean ± SEM of beam breaks recorded for 30 min. Statistical analysis of the results was conducted using one-way analysis of variance (ANOVA), followed by Dunnett’s test. Significant difference was compared to the vehicle-treated group: **p < 0.01, ***p < 0.001, *N* = 8

Four of the tested compounds (**4**, **5**, **8**, and **9**), as well as lacosamide, did not significantly influence on spontaneous locomotor activity in mice. Compound **15** reduced the number of crossings registered with photoresistor actometers by 29% but not in a statistically significant way. Strong impact on the behavior of mice in this test was recorded for agent **16**, which inhibited the locomotor activity in studied animals by 86% (*p* < 0.001). Moreover, the first-generation anticonvulsant drug—valproic acid—at the tested dose demonstrated sedative properties, since it significantly decreased locomotor activity in mice by 62%. On the other hand, the second-generation anticonvulsant drug lacosamide had no significant influence on locomotor activity. The obtained results are presented in Fig. 3.

### Antiallodynic activity in the oxaliplatin-induced neuropathy

**Fig. 4:**
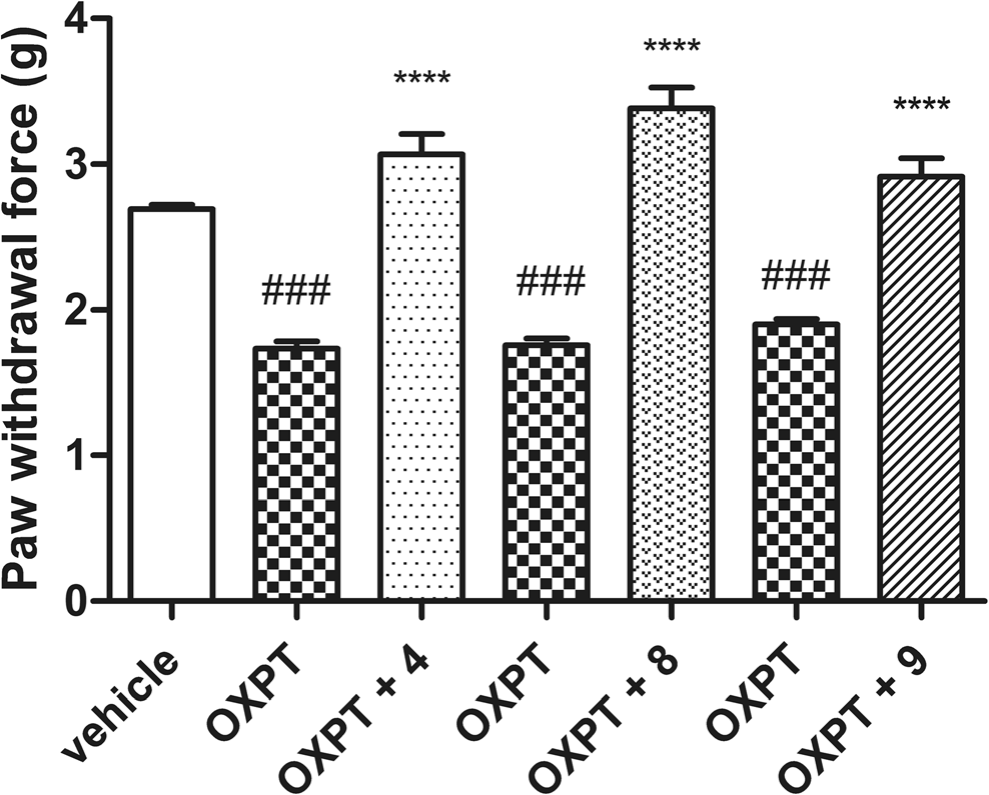
Effect of selected compounds **4**, **8**, and **9** on tactile allodynia in oxaliplatin-treated neuropathic mice assessed 7 days after oxaliplatin injection measured using von Frey test. Data are presented as mean ± SEM. Statistical analysis of the results was conducted using repeated measures ANOVA, followed by Bonferroni’s multiple comparison test. Allodynic effect of oxaliplatin-treated vs. vehicle-treated mice: ^###^ *p* < 0.001. Antiallodynic effect of the tested compounds vs. oxaliplatin-treated mice: ****p < 0.0001, *N* = 9–10. *OXPT* oxaliplatin

In the view of mentioned results, three compounds (**4**, **8**, and **9**) with significant analgesic activity in the formalin model and without sedative properties were also examined in the oxaliplatin-induced neuropathic pain. Mechanical allodynia in oxaliplatin-treated mice was measured 7 days after injection. The mean force that caused paw withdrawal was 2.69 ± 0.03 g in the control group (animals not treated with oxaliplatin), whereas this value range from 1.74 ± 0.05 to 1.88 ± 0.04 g, respectively, in the group of oxaliplatin-treated animals. As it is shown in Fig. 4, in neuropathic animals, all tested compounds (**4**, **8**, and **9**) attenuated tactile allodynia, since they significantly elevated the pain sensitivity threshold by 78, 92, and 58%, respectively (*p* < 0.0001 in all groups). Previous research from our laboratory demonstrated that pregabalin given at the dose of 30 mg/kg elevated pain sensitivity threshold by 122% (*p* < 0.001) in neuropathic mice (Sałat et al. 2014).

### Influence on memory in the passive avoidance step-through task

**Fig. 5:**
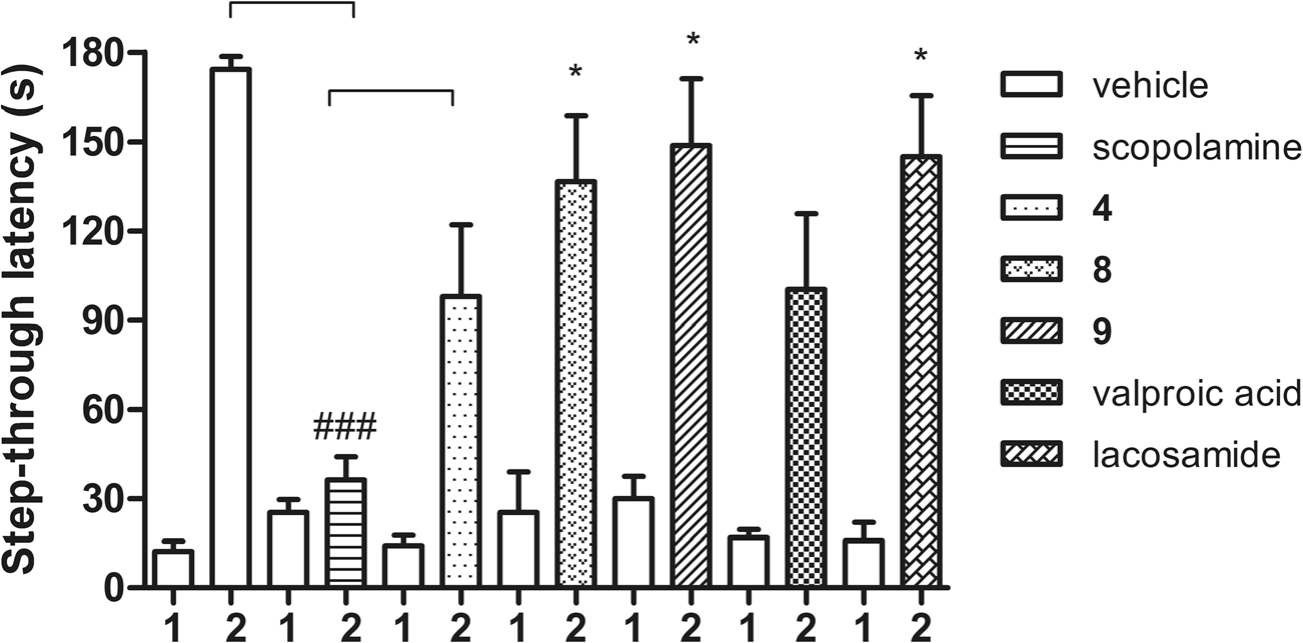
Effects of compounds **4**, **8**, **9**, valproic acid, lacosamide, and scopolamine on learning and memory in the passive avoidance test. Data are presented as mean ± SEM in the acquisition phase (day 1) and in the retention phase (day 2). Statistical analysis of the results was conducted using Kruskal-Wallis test, followed by Dunn’s multiple comparison test. Significant difference was compared with the vehicle-treated mice: ^###^ *p* < 0.001. Significant difference was compared with the scopolamine-treated mice: *p < 0.05, ***p < 0.001, *N* = 8–10

The effect of three selected compounds **4**, **8**, and **9**, as well as valproic acid and lacosamide, at the doses corresponding to their ED_50_ values from the MES test, on memory, was investigated in the step-through passive avoidance test. In this test, scopolamine, a well-known muscarinic antagonist, which interferes with memory in animals and humans, was used as a positive control (Kwon et al. 2010). In the acquisition phase, the step-through latency was similar in all experimental groups. In the retention phase of this test, all tested and reference compounds prolonged step-through latency time in mice (vehicle group: from 12.2 ± 3.4 to 174.2 ± 4.3 s; scopolamine: from 25.3 ± 4.3 to 36.2 ± 7.9 s; **4**: from 14.1 ± 3.4 to 97.9 ± 24.2 s; **8**: from 26.7 ± 12.0 to 123.9 ± 23.1 s; **9**: from 29.9 ± 7.6 to 148.8 ± 22.2 s; valproic acid: from 16.9 ± 2.7 to 100.3 ± 25.5 s; lacosamide: from 15.7 ± 6.2 to 145.0 ± 20.4 s) (Fig. 5). In the scopolamine-treated group, the prolongation of latency time was significantly shorter compared to that of vehicle-treated mice (*p* < 0.001). In turn, compounds **8** and **9** and lacosamide significantly prolonged step-through latency time compared to the scopolamine-treated mice (*p* < 0.05 for all), whereas for compound **4** and valproic acid the results were not statistically significant.

### Anticonvulsant activity in the 6-Hz test

The initial anticonvulsant screening of the tested compounds in the 6-Hz test was performed at a fixed dose of 100 mg/kg. The obtained results revealed that all compounds demonstrated protection in half or more of the animals. Compounds **4** and **8** showed prolonged antiseizure activity (at least of 50% protection) at four time points, whereas compounds **5** and **9** displayed antiseizure activity at three time points (0.25, 0.5, and 1 h). Shorter lasting anticonvulsant effect was observed for compounds **15** and **16**, as they showed protection at 0.25 and 0.5 h (Table 2). Based on the preliminary results for tested compounds, their median effective doses (ED_50_) in the 6-Hz test were determined at time point of their peak antiseizure activity. The obtained ED_50_ values, ranging from 24.66 (compound **4**) to 47.21 mg/kg (compound **16**), are presented in Table 3. Summarizing, in the 6-Hz test, the examined compounds revealed higher activity than valproic acid but lower than lacosamide.

**Table 2:** Anticonvulsant activity of the tested compounds in the 6-Hz test following i.p. administration in mice (dose of 100 mg/kg)

Cmpd	6-Hz test			
	0.25 h	0.5 h	1 h	2 h
**4**	*3/4*	*3/4*	*3/4*	*2/4*
**5**	*4/4*	*4/4*	*3/4*	0/4
**8**	*4/4*	*4/4*	*2/4*	*2/4*
**9**	*4/4*	*4/4*	*2/4*	0/4
**15**	*4/4*	*3/4*	0/4	0/4
**16**	*2/4*	*4/4*	0/4	0/4

Data indicate number of mice protected/number of mice tested. Ratios where at least two animals were protected have been highlighted in italics for easier data interpretation. The animals were examined at four pretreatment times: 0.25, 0.5, 1, and 2 h

**Table 3:** The quantitative pharmacological parameters ED_50_ values in the 6-Hz test following i.p. administration in mice

Compound	TPE (h)	ED_50_ (mg/kg)
**4**	0.25	24.66 (18.13–33.55)
**5**	0.25	31.97 (19.66–51.98)
**8**	0.25	33.79 (12.79–89.29)
**9**	0.25	40.49 (24.26–67.58)
**15**	0.25	38.75 (23.48–63.94)
**16**	0.5	47.21 (34.29–65.02)
Valproic acid	0.5	130.64 (117.61–145.19)
Lacosamide	0.5	5.25 (3.55–7. 76)

*TPE* time to peak effect

### Binding studies

In order to better understand the plausible molecular targets by which tested compounds might act, for a selected agent (**4**), the binding assays for sodium channel (site 2), L-type calcium (dihydropyridine and verapamil sites), as well as for N-type calcium channels were carried out using [^3^H]batrachotoxin, [^3^H]nitrendipine, [^3^H]D888, and [^125^I]*ω*-conotoxin GVIA, as radioligands, respectively. Moreover, the binding studies for NMDA, GABA_A_ and neuronal α4β2 nicotinic receptors were performed. Compound **4** only at the high concentration of 500 μM revealed the effective binding to the neuronal sodium channels (site 2), as it is indicated by the inhibition greater than 50%. At this concentration, it also revealed moderately influence on L-type calcium channels (verapamil site), 45%, as well as did not bind to L-type calcium (dihydropyridine site) and N-type calcium channels. At a concentration of 200 μM, the tested molecule revealed also moderate affinity to NMDA receptors (antagonist radioligand). Compound **4** at a concentration of 100 μM did not bind effectively to sodium (site 2), L-type calcium (dihydropyridine and verapamil sites), and N-type calcium channels as well as GABA_A_ and neuronal α4β2 nicotinic ionotropic receptors. The binding results are shown in Table 4.

**Table 4:** In vitro binding assays for compound **4**

Assay	Ligand	Concentration [μM]	% Inhibition of control specific binding
Na^+^ channel site 2 (antagonist radioligand)	[^3^H]batrachotoxinin	100	15.9
		500	*56.0*
L-type Ca^2+^ (dihydropyridine site) (antagonist radioligand)	[^3^H]nitrendipine	100	−14.8
		500	0.7
L-type Ca^2+^ (verapamil site) (antagonist radioligand)	[^3^H]D888	100	15.0
		500	*45.0*
N-type Ca^2+^(antagonist radioligand)	[^125^I]ω-conotoxin GVIA	100	0.2
		500	0.9
NMDA (antagonist radioligand)	[^3^H]CGP 39653	100	15.0
		200	*35.0*
GABAA1 (alpha 1,beta 2,gamma 2) (agonist radioligand)	[^3^H]muscimol	100	−18.3
N neuronal alpha4beta2 (agonist radioligand)	[^3^H]cytisine	100	3.0

Results showing an inhibition higher than 50% are considered to represent significant effects of the test compounds; results showing an inhibition between 25 and 50% are indicative of moderate effect; results showing an inhibition lower than 25% are not considered significant

Significance and moderate effects have been highlighted in italics for easier data interpretation

### Potential hepatotoxicity activity

**Fig. 6:**
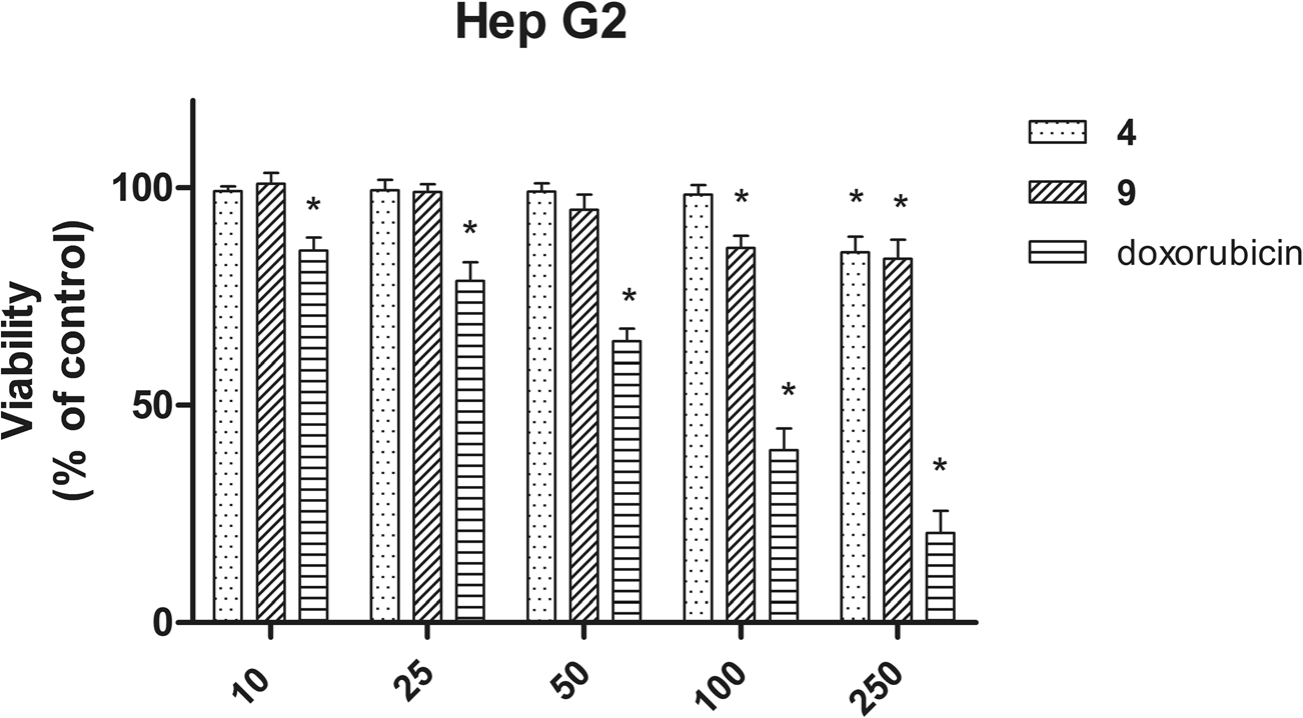
Viability of HepG2 cells incubated in the presence of selected compounds in concentration range 10–250 μM for 24 h. The graph shows results from MTT assay expressed as the percentage of control condition ± SEM. Three independent experiments were performed. Doxorubicin was used as positive control: *p < 0.05

The MTT assay revealed that both tested compounds (**4** and **9**), at a concentration range of 10–50 μM, were safe (cell viability: above 90%); only slight cytotoxic effects appeared at higher concentrations of tested compounds (100 and 250 μM), but cell viability was still high (above 80%). In addition, cytotoxicity effect of chemotherapeutic agent—doxorubicin (anthracycline antibiotics)—on Hep G2 cells was evaluated as positive control at the same concentration as evaluated compounds (Fig. 6). Strong cytotoxic effect on Hep G2 cells of doxorubicin was observed even at low concentrations (under 50 μM). Performed experiments show that both compounds (**4** and **9**) did not induce hepatotoxic effects.

## Discussion

Hybrid molecules are suggested to be a more effective and safer remedy for treatment of various diseases than combination therapy (Kleczkowska et al. 2016). Our previous research in the group of new hybrid molecules, which join on the one chemical template the structural fragments of well-known anticonvulsant drugs such as ethosuximide (effective in the scPTZ seizures), levetiracetam (active in the 6-Hz test), and lacosamide (active in the MES and the 6-Hz tests), proved that a number of these agents revealed prominent anticonvulsant properties and broad spectrum of activity in animal models of seizures, i.e., MES, scPTZ, and 6-Hz tests (Kamiński et al. 2015a, b, 2016a). Furthermore, some derivatives of pyrrolidine-2,5-dione demonstrated analgesic and antiallodynic properties in the formalin model of tonic pain and in oxaliplatin-induced neuropathy (Kamiński et al. 2016b; Obniska et al. 2015a, 2016; Rapacz et al. 2016a, b). Therefore, and as well as because of the increasing role of anticonvulsant drugs in the treatment of pain, the first aim of the present study was evaluation of antinociceptive activity of six selected hybrid amides derived from *N*-benzyl-2-(2,5-dioxopyrrolidin-1-yl)propanamide and 2-(2,5-dioxopyrrolidin-1-yl)butanamide, based on the previously obtained preliminary results (Kamiński et al. 2015a). Among these compounds, four of them, **4**, **8**, **15**, and **16**, demonstrated anticonvulsant activity in the MES and the scPTZ tests, whereas two of them, **5** and **9**, were active only in the MES test. All these compounds revealed substantial safety profiles in the rotarod test for acute neurological toxicity (TD_50_ >300 mg/kg (**4**, **5**, **8**, **15**, and **16**) and TD_50_ >500 mg/kg (**9**), i.p., mice) that provided distinctly better protective indices compared to model anticonvulsant drugs (carbamazepine, ethosuximide, and valproic acid) and similar to that obtained for lacosamide (Kamiński et al. 2015a).

It has been reported in preclinical studies that numerous anticonvulsant active agents which are active in the MES test exert an antinociceptive action in the animal models of pain (Löscher and Schmidt 2011; Obniska et al. 2015a, b). Therefore, in the present study, antinociceptive activity of the tested compounds were examined at the dose which was its median effective dose (ED_50_) determined in the MES test. At the same dose, their influence on spontaneous locomotor activity and contextual memory was also checked. At first, analgesic activity was investigated in the model of acute pain—the hot plate test. However, both the test and the reference compounds, examined at the anticonvulsant active doses, demonstrated no significant analgesic properties in this model of acute pain. In the previous study, we reported that lacosamide tested at higher dose—30 mg/kg—revealed significant antinociceptive effect (Rapacz et al. 2016a, b). In the next step, all compounds were also examined in the formalin test of tonic pain. In the first (neurogenic) phase of the test, four compounds, **4**, **8**, **9**, and **16**, as well as valproic acid and lacosamide statistically significantly attenuated the nocifensive response. In the second phase of the formalin test, a very prominent antinociceptive activity demonstrated compounds **4**, **5**, **8**, and **9**,as well as reference anticonvulsant agents. Also in other studies, anticonvulsant drugs, including lacosamide and tiagabine, revealed analgesic activity in both phases of the formalin test (Beyreuther et al. 2006; Laughlin et al. 2002; Rapacz et al. 2016b; Stöhr et al. 2006). On the other hand, lamotrigine and gabapentin inhibited only the late phase formalin behaviors (Laughlin et al. 2002). Recently, it has been reported that valproic acid at a dose of 300 mg/kg showed a significant reduction in the acute and inflammatory phases , but at a dose of 100 mg/kg, it reduced significantly the licking time only in the inflammatory phase (Kaufmann et al. 2016). The first phase of the formalin test is connected with acute chemical pain, whereas the second one is defined as tonic nociception involving central sensitization of dorsal horn neurons of the spinal cord or inflammation-induced hyperactivity of afferent nociceptors or combination of both (Laughlin et al. 2002; Ximenes et al. 2013). Thus, it is suggested that anticonvulsant drugs have more considerable effect on the inhibition of sensitized signaling than on normal transient nociceptive signaling (Laughlin et al. 2002).

Regretfully, all known anticonvulsant drugs often produce adverse effects, which range from mild disturbances of CNS functions to serious cases of liver insufficiency or bone marrow damage (Lasoń et al. 2011). To evaluate whether compounds possess the ability to induce CNS depression, and in this way influence antinociceptive effect, the spontaneous locomotor activity was measured. Compounds **4**, **5**, **8**, **9**, **15**, and lacosamide at the tested doses did not exhibit sedative properties, whereas compound **16**, as well as valproic acid, significantly diminished locomotor activities.

Subsequently, selected compounds **4**, **8**, and **9**, which revealed prominent antinociceptive activity in the formalin test as well as without sedative properties, were examined to establish their antiallodynic efficacy in the model of painful peripheral neuropathy induced by a chemotherapeutic drug—oxaliplatin (Ling et al. 2008). Many reports have indicated that gabapentinoids significantly inhibited allodynia induced by oxaliplatin or paclitaxel (Aoki et al. 2014; Gauchan et al. 2009; Xiao et al. 2007). In the present study, compounds **4**, **8**, and **9** demonstrated a prominent elevation of mechanical nociceptive threshold in oxaliplatin-treated mice. All tested compounds significantly reduced mechanical hypersensitivity and completely reversed oxaliplatin-induced allodynia in the von Frey test. Previous research from our laboratory demonstrated that pregabalin, which is used in the treatment of neuropatic pain, strongly and significantly elevated pain sensitivity in neuropathic mice (Sałat et al. 2014).

Most commonly used antiepileptic drugs (including phenytoin and valproic acid) worsen learning and memory in the patients with epilepsy (Cloyd et al. 2006). On the other hand, the change in pharmacokinetics and higher sensitivity to adverse effects of many antiepileptic drugs associated with aging generally necessitate more cautious selection of drugs and dosing in elderly people (Schmidt and Schachter 2014). Thus, it is important in preclinical studies to control possible CNS adverse effects, like diminished attention, language skills, memory, and processing speed. Herein, to assess the effects of the tested compounds on learning and memory, a passive avoidance test was used. Compounds **8** and **9** and a new-generation anticonvulsant drug—lacosamide—did not cause cognitive deficits, as they significantly prolonged step-through latency time compared to the scopolamine-treated mice. In the case of compound **4** and first-generation anticonvulsant drug, valproic acid, this prolongation of the latency time was lower and not statistically significant.

Data on hepatotoxicity of some antiepileptic drugs, including valproic acid (Nanau and Neuman 2013), lamotrigine (Su-Yin et al. 2008), and pregabalin (Sendra et al. 2011), became the premise for determination of potential hepatotoxicity of selected compounds possessing anticonvulsant activity. Therefore, selected compounds **4** and **9** were evaluated for potential cytotoxicity against human cancer cells (Hep G2). Performed experiments showed that tested compounds did not induce cytotoxic effect on hepatoma cells.

The second aim of the present study was to extend anticonvulsant studies of selected molecules using the 6-Hz test. Psychomotor seizures induced by a 6 Hz stimulation is deemed to be a model of therapy-resistant partial seizures (Barton et al. 2001). The model reference drug, levetiracetam, which is highly active in this model (ED_50_ = 15.73 mg/kg) (Rapacz et al. 2016a), does not protect rodents against seizures in the MES and scPTZ tests up to doses of 500 mg/kg—the most widely used tests in the preclinical studies (Löscher and Schmidt 2011; Schmidt and Schachter 2014). Therefore, the 6-Hz test is proposed to be used routinely, apart from MES and scPTZ tests to screen numerous novel molecules in preclinical studies. In the present study, all of tested new hybrid agents (**4**, **5**, **8**, **9**, **15**, and **16**) displayed anticonvulsant properties in the psychomotor seizures test with ED_50_ values ranging from 24.66 to 47.21 mg/kg.

Current clinically potent anticonvulsant drugs affect various molecular targets, including modulation of voltage-gated ion channels, enhancement of GABAergic transmission, blockade of ionotropic glutamate (NMDA, AMPA) receptors, or interactions with elements of the synaptic release machinery (SV2a protein), and most have more than one mechanism of action (Lynch et al. 2004; Klitgaard et al. 2016). It is worth mentioning that regardless of the mechanism of action, they all act to reduce hyperexcitability by either decreasing excitatory or enhancing inhibitory neurotransmission (Löscher et al. 2016). Voltage-dependent sodium and calcium channels seem to play a crucial role in establishing and regulating the excitability of CNS nerves and are the most common targets among currently available anticonvulsant drugs, including phenytoin, carbamazepine, lamotrigine, oxcarbazepine, and lacosamide (Brodie et al. 2011; Liu et al. 2003; Mantegazza et al. 2010). Interestingly, lacosamide, in contrast to the aforementioned drugs, enhances the slow inactivation of sodium channels without affecting the fast inactivation (Rogawski et al. 2015). The anticonvulsant drugs which influence the activity of high voltage-activated calcium channels are phenytoin, felbamate, topiramate, lamotrigine, and levetiracetam (Lukyanetz et al. 2002; Meldrum and Rogawski 2007; Shank et al. 2000), whereas gabapentin and pregabalin interact with the α_2_δ-1 subunit of neuronal voltage-dependent calcium channels (Rogawski and Löscher 2004). Valproic acid is associated with several mechanisms of action, including GABA potentiation, glutamate (NMDA) inhibition, sodium channel, and T-type calcium channel blockade (Klitgaard et al. 2016). This drug is widely used as anticonvulsant in partial and generalized convulsive seizures and absence seizures as well as in migraine prophylaxis. Moreover, recent evidence points also to the involvement of neuronal nicotinic receptors in epilepsy and pain sensation (Fonck et al. 2005; Lloyd and Williams 2000). A numerically abundant nicotinic receptor subtypes in the CNS are α4β2, α3β4, and α7 (Lukas et al. 1999). It has been reported that carbamazepine and oxcarbazepine were found to block neuronal α4β2 nicotinic receptors, which may explain their particular efficacy in nocturnal frontal lobe epilepsy that can be caused by mutant neuronal nicotinic receptors (Di Resta et al. 2010). Taking into account the previous remarks, for selected compound **4**, its influence on sodium channel (site 2) and L-type and N-type calcium channels, as well as for NMDA, GABA_A_, and neuronal α4β2 nicotinic receptors, was performed. The results from the binding studies showed that compound **4** only at the highest concentration of 500 μM revealed the effective binding to the neuronal sodium channels (site 2) and moderate binding to the verapamil site of L-type calcium channels. Moreover, at concentration of 200 μM, it binds moderately with NMDA receptors. However, it should be stressed that the pharmacokinetic studies for compound **4** have not been carried out and thus the pharmacologically active in vivo concentration of this molecule is not known. Therefore, the in vitro binding studies enable only the preliminary and tentative determination of the mechanism of action. It is of interest to note that carbamazepine, a well-known anticonvulsant drug that acts as sodium blocker, showed also only a moderate effect on sodium channels at concentration 500 μM (33.6% inhibition of control specific binding) (Kamiński et al. 2016b). Bearing in mind very promising anticonvulsant and analgesic properties of the tested hybrid molecules, as well as the fact that currently available anticonvulsant drugs act by a variety of mechanisms, further detailed studies in terms of plausible molecular targets should be performed, especially including the pharmacokinetic properties of the aforementioned substances.

## Conclusions

The results obtained in the present study indicate that some of novel hybrid molecules derived from *N*-benzyl-2-(2,5-dioxopyrrolidin-1-yl)propanamide and 2-(2,5-dioxopyrrolidin-1-yl)butanamide displayed prominent analgesic effects in animal models of pain. In both phases of formalin test, antinociceptive activity demonstrated compounds **4**, **8**, and **9**. These compounds relieved also mechanical allodynia in oxaliplatin-induced neuropathic pain model. It is noteworthy that at active doses, no sedative properties were recorded for these compounds as well as lacosamide. Moreover, for compounds **8** and **9** and lacosamide, no deleterious effect on memory was observed, while compound **4** and valproic acid might induce memory deficits. Additionally, in the MTT assay, tested compounds (**4** and **9**) did not induce cytotoxic effect on hepatoma cells. The extended anticonvulsant studies proved that all tested compounds (**4**, **5**, **8**, **9**, **15**, and **16**) inhibited psychomotor seizures in the 6-Hz test.

Summing up, our previous and present preclinical results proved that these novel hybrid molecules demonstrate very promising anticonvulsant and analgesic activity. Compounds **4** and **9** displayed not only a broad spectrum of anticonvulsant activity but also collateral prominent analgesic and antiallodynic properties.
